# Young Adults With Developmental Coordination Disorder Adopt a Different Visual Strategy During a Hazard Perception Test for Cyclists

**DOI:** 10.3389/fpsyg.2021.665189

**Published:** 2021-04-14

**Authors:** Griet Warlop, Pieter Vansteenkiste, Matthieu Lenoir, Frederik J. A. Deconinck

**Affiliations:** Department of Movement and Sports Sciences, Ghent University, Ghent, Belgium

**Keywords:** developmental coordination disorder, hazard perception, cycling, traffic safety, gaze behavior, young adults

## Abstract

Cycling in traffic requires a combination of motor and perceptual skills while interacting with a dynamic and fast-changing environment. The inferior perceptual-motor skills in individuals with developmental coordination disorder (DCD) may put them at a higher risk for accidents. A key skill to navigate in traffic is to quickly detect hazardous situations. This perceptual-cognitive skill was investigated in young adults with DCD using simulated traffic situations in a hazard perception test in cycling. Nine individuals with DCD (age: 23.0 ± 3.8) and nine typically developing (TD) individuals (age: 24.6 ± 3.5) participated in the study and completed the test while their gaze was tracked using a remote eye tracking device. A questionnaire was used to determine cycling experience and the perception of cycling and anticipation skill in traffic. Despite a longer period to master the motor skill of cycling, individuals with DCD reported to be able to safely cycle in traffic around the same age as TD young adults. In the hazard perception test, individuals with DCD fixated the hazards later, less frequently and for a shorter duration than the TD participants, however, the participants with DCD did not wait longer to react to the hazard than the TD participants. Interestingly, individuals with DCD rated the traffic situations in the test as significantly more dangerous than the TD participants. In conclusion, the differences exposed in the hazard perception test may imply an increased risk of accidents in individuals with DCD. In further research and practice it is recommended that both the motor and the perceptual aspects of cycling are addressed.

## Introduction

Navigating safely through traffic, whether as a car driver, cyclist or pedestrian, depends on cognitive, and perceptual-motor processes. Irrespective of the transport mode, it is important to correctly assess the situation at any time. According to Endsley’s (1995) concept of “situational awareness” this entails three levels: the perception of the environment and events with respect to time and space, comprehension of their meaning and projection of their future states. It is clear that what at first glance occurs unconsciously and automatically is actually a very complex task. Hence, it is not surprising that individuals who experience problems with aspects of these cognitive or perceptual-motor processes (e.g., ADHD or ASD) also have difficulty assessing traffic situations (Clancy et al., 2006; Cowan et al., 2018; Wilmut and Purcell, 2021). A group that deserves the necessary attention in this regard is individuals with developmental coordination disorder (DCD). DCD is an idiopathic neurodevelopmental disorder characterized by significant impairments in motor coordination and learning.

Individuals with DCD have difficulties with perceptual function (for review see Wilson and McKenzie, 1998), oculomotor function (Warlop et al., 2020), executive function (Tsai et al., 2012), and forward modeling (for review see Wilson et al., 2013) all of which are core abilities within the situational awareness model. For example, when it comes to level 1 (perception of the environment and events) Purcell et al. (2012) demonstrated reduced looming sensitivity in children with DCD when observing cars approaching as a pedestrian. This deficit may then lead to choosing inadequate crossing gaps, as found in a follow-up study of Purcell et al. (2017), indicating lack of comprehension of the meaning of the perceptual input (level 2) and/or underlying problems with projection of future states (level 3). It is also worth noting that individuals with DCD are found to have reduced working memory capacity (Alloway, 2011). This may result in lower performances in high cognitively demanding tasks like a hazard perception test, as shown by Wood et al. (2016). Remarkably, recent research shows that both children and adults with DCD perceive road crossing, as a pedestrian, as a more challenging task than typically developing (TD) peers (Wilmut and Purcell, 2020), which indicates that the individuals are to some extent aware of the risk associated with their perceptual-motor problems. In this respect, in this important to highlight that individuals with DCD also perceive themselves as less competent car drivers and avoid active participation to traffic (Kirby et al., 2011a). Ultimately, individuals with DCD may end up in a negative spiral, given that experiential learning is essential in the education of situational awareness.

In the current study, we build upon this line of research and examine individual’s with DCD ability to perceive hazards in traffic while cycling. Hazards in traffic, especially when cycling, are ubiquitous and can be both static (e.g., curbs and potholes) and dynamic (approaching cars or pedestrians crossing the road). By definition, hazard perception involves the three levels of the situational awareness model (Wetton et al., 2011; Vansteenkiste et al., 2016), and therefore requires adequate perception, recognition, and projection of the environment and events. Previous research has shown that gaze behavior (i.e., visual search) is a crucial factor in this process. For example, Zeuwts et al. (2016) showed that young learner cyclists fixate the hazards later, and have slower reaction times than experienced adult cyclists. Also in car driving, effective hazard perception performance appears to depend on how quickly the hazards were fixated (Crundall et al., 2012). Furthermore, when cognitive load is increased, individuals with low working memory capacity fixate less on the hazards, resulting in slower reactions to hazards (Wood et al., 2016).

Perception of hazards is quintessential to ensure safety to the individual, and hence, it is important to have insight into the performance of individuals with DCD in this matter, whom we know have underlying deficits that may put them at risk. This will be investigated with a standardized hazard perception test, in which both gaze behavior and reaction (time) are examined. Based on previous reports, e.g., on reduced looming sensitivity or forward modeling, and consistent with findings of immature gaze behavior in children, we expect less efficient visual search with later fixation and longer reaction times in individuals with DCD. Given the critical role of the perception of risk and the perceived competence of cycling skill in the individuals’ decision to actively engage in traffic these factors were also documented.

## Materials and Methods

### Participants

Eighteen adults aged between 19 and 30 years old participated in the current study. Nine of these participants were clinically diagnosed with DCD as a child by a pediatrician and recruited via social media and a database of participants that were involved in previous studies (Deconinck et al., 2006a, b). One participant with DCD was excluded after testing due to insufficient tracking accuracy in the HP-test (details of the included participants shown in Table 1). The control group, recruited via convenience sampling, consisted of nine TD individuals who have never been diagnosed with a neurodevelopmental disorder or medical condition that could affect motor behavior. All participants with DCD complied to the diagnostic criteria as described in the Diagnostic and Statistical Manual of Mental Disorders (DSM-5; American Psychiatric Association, 2013). For example, their motor skills were below that expected according to their age (criterion A). This was assessed, as part of this study, with the Movement Assessment Battery for Children-2 (MABC-2; Henderson et al., 2010), which is designed and norm-referenced up to the age of 16. This test battery discriminated between poor and normal motor competence in previous studies in young adults with DCD (Wilmut et al., 2013; Du et al., 2015), and was therefore considered suitable for this study. Age band 3 and the reference values of the 16 olds were used to determine the participants’ percentile scores. Two participants with DCD scored at the 25th percentile, which is above the cut-off value for “at risk for DCD.” However, they both scored high on the Adult DCD Checklist (ADC; Kirby and Rosenblum, 2008; Kirby et al., 2011b), which assessed past motor difficulties in childhood (section 1 of the checklist; DSM criterion C) and current daily motor functioning (section 2 of the checklist; DSM criterion B). A score of 17 or higher on the first section of this checklist is indicative for “probable DCD.” In the TD group, all participants scored at or above the 25th percentile of the MABC-2 and had a maximum score of 3 on the first section of the ADC. None of the participants reported to have neurological conditions affecting movement (other than DCD in the DCD group; DSM criterion D), and they all had normal or corrected-to-normal vision. The study was approved by the Ethics Committee of the Ghent University Hospital and conducted in accordance with the Declaration of Helsinki.

**TABLE 1:** Summary of the participant’s characteristics (mean ± SD).

	DCD (*N* = 8)	TD (*N* = 9)
Gender (number of participants)	3 male 5 female	3 male 6 female
Age (year)	23.0 ± 3.8	24.6 ± 3.5
Dominant hand (number of participants)	4 left 4 right	2 left 7 right
MABC-2 percentile	9.3 ± 11.0	68.9 ± 22.8
ADC score section 1	22.9 ± 3.9	1.4 ± 1.1
ADC score section 2	53.1 ± 16.9	10.1 ± 8.6

### Apparatus

#### Cycling Experience and Perceived Cycling Skills Questionnaire

To get a better understanding of the participant’s cycling experience, the following cycling milestones were assessed: the age at which the participants started to learn how to ride a bike, the age at which they mastered the motor skill of independently riding a bike, and the age that they were able to safely cycle in traffic. Also, the participants’ total years of cycling experience and how frequently they cycle was surveyed. To get an indication on the participants’ perception of their own cycling skills the following three questions were added to the questionnaire: a yes-no question on if they thought they could safely cycle in traffic, and two 5-point Likert scale questions assessing how well they perceived their cycling ability and how well they anticipated hazards in traffic (ranging from “not good” to “very good”). From all questionnaire measures, raw data were used for statistical analysis.

#### Hazard Perception Test

For the development of the HP-test, videos of real-life traffic situations were recorded by cycling through traffic in two Flemish cities with a GoPro Hero3 (30 Hz, full HD and 170° field of view) mounted on a helmet. Some traffic scenarios were staged using volunteers to safely create hazardous situations in calm streets. The recordings were made only on straight streets while constantly looking forward and not turning the head towards specific objects, side streets or other road users. Recordings with head movements were excluded. All clips were stabilized to reduce vibrations resulting from the state of the bicycle path or small head movements using dedicated video stabilization software “Mercalli V2” (ProDad). For this study, a selection of 14 video fragments with a duration of 10–50 s was made. The videos were filmed from the cyclist’s point of view and all contained at least one hazard (nine videos contained one hazard, two contained two hazards, and one contained four hazards). A hazard was defined as a traffic situation which exposes the cyclist to an increased possibility of an accident and makes the cyclist brake or change direction in order to avert this accident. Both behavioral predication (BP) hazards (i.e. objects or road users that are already visible prior to developing as a hazard), and environmental prediction (EP) hazards (i.e. potentially hazardous situations that are not visible before the actual hazard occurs, yet, are inferable from other objects than the one causing the hazard) were included (Crundall et al., 2012). See the Supplementary Material for an example fragment and Supplementary Table 1 for a description and details of the included video clips and hazards. This type of test was used by Vansteenkiste et al. (2016) and Zeuwts et al. (2016) and proved useful in testing differences between adults and children. The HP-test was carried out with the Remote Eye Tracking Device (RED) of SensoMotoric Instruments (Teltow, Germany), which registered the participant’s gaze during the test. The video fragments were shown on a 22-inch computer screen underneath which the eye tracking device was mounted. Two beams of infrared light illuminated the eyes and the reflections on the cornea were captured by an infrared camera to determine the position of the pupils and hence the direction of the gaze. The system has a manufacturer-reported accuracy of 0.4°. A laptop, running the Experiment Center 3.4 software, was connected to the device and recorded the gaze data at a binocular sampling rate of 120 Hz.

### Procedure

Prior to the HP-test, the participants filled in a short questionnaire on their cycling experience and how they perceived themselves as a cyclist. Then, they were asked to take place in front of the screen equipped with the RED. Their position was adapted so that the distance between their eyes and the screen was between 60 and 80 cm, resulting in a visual angle ranging between 24 and 32 degrees (vertical), 31–41 degrees (horizontal). Once the participant was seated comfortably and the device was capturing their eye well, a 5-point calibration was done. When the calibration did not result in an accuracy below 0.6° it was repeated. If this accuracy was not achieved after five trials of calibration, the test was continued with the best possible calibration. Although the RED is quite resistant for small movements, the participants were asked to stay in the same position throughout the experiment to assure good recording of the gaze behavior. At the end of the test, a calibration check was done. The participants were instructed to observe the videos and imagine that they were cycling themselves. They were asked to click the mouse when they would use the brakes, change direction or stop for a hazard. After each fragment the participants were asked how safe the traffic scenario was from the perspective of the cyclist on a 5-point Likert scale ranging from “not hazardous” to “very hazardous.” A total of 14 videos with a duration of 20–30 s were displayed, resulting in a total test duration of 10–15 min for the HP-test.

### Analysis

#### Gaze Behavior

Prior to quantitative analysis, the quality of the gaze data was assessed. Data of one participant with DCD was deleted, due to insufficient tracking accuracy (8.61°) throughout the test. The averaged accuracy of the included data was 0.74 ± 0.33°. Second, the tracking ratio, which is the percentage of time that eye movements were effectively measured, was evaluated. Trials were excluded when tracking ratio was lower than 80% (Vansteenkiste et al., 2016). For this reason, nine trials (over three participants) were excluded from further analysis. In addition, one video clip was excluded in all participants as none of the participants reacted to the hazard, so it did not seem to be perceived as hazardous by any of the participants. Finally, in one video the hazard was detectable from the very start of the fragment, which resulted in very different reaction times across the subjects. It was unclear what information or which cues led to the responses, so it was decided to exclude this fragment as well, resulting in 12 video clips included in the statistical analysis. In BeGaze 3.7 (SensoMotoric Instruments, Teltow, Germany), fixations were determined using the SMI fixation detection algorithm. In each video clip the hazards (specified in Supplementary Table 1) were determined and indicated as Areas Of Interest (AOI) using the dynamic AOI editor. The AOI’s were polygons around the hazards that changed shape and size dynamically along with the movement and looming of the hazard in the video clip. Then, the number of fixations, the duration of the fixations, the duration of the first fixation on the AOI, dwell time (i.e., the total time spent fixating on an AOI), and the timing of the first fixation on the hazard relative to the appearance of the hazard, were calculated per AOI. As the nature and the duration of the traffic situations and the hazards varied between the fragments, *z*-scores were calculated of all gaze behavior measures using the means (M) and standard deviation (SD) of the TD control group per AOI: $z=\frac{Rawscore-M_{TD}}{SD_{TD}}$. Finally, for each gaze behavior variable, the average of the *z*-scores of all AOI’s was calculated.

#### Response Rate and Reaction Time

Response rate, which referred to the number of hazards that the participants clicked for within the time interval that a hazard was visible on the video clip, was counted and expressed in relation to the total number of hazards. In addition, extra clicks, i.e., clicks before or after the time interval related to the hazard, or additional clicks within this time interval, were summed across all trials. Reaction time was measured in ms from the first appearance of the hazard. As different hazards had different lengths of intervals during which the hazard was visible, reaction time was strongly dependent on the nature of the video. Therefore, reaction times per AOI were also converted into *z*-scores in a similar way to the gaze behavior metrics and the average of the *z*-scores of all AOI’s was calculated.

#### Statistics

To assess the criteria for parametric testing, Kolmogorov–Smirnov tests were conducted for normality and Levene’s tests were performed to assess the homogeneity of variance. For normally distributed data with equal variances, independent samples *T*-tests were carried out to investigate differences between TD and DCD on all variables. In the instance of not normally distributed data, non-parametric Mann–Whitney U tests were conducted. In the instance of unequal variances, Welch’s corrections were applied. The alpha level was set at 0.05 and effect sizes were reported as Cohen’s *d*, which was calculated as: $d=\frac{M_{DCD}-M_{TD}}{SD_{TD}}$. Indicative thresholds for Cohen’s *d* are small (0.2), medium (0.5), and large (0.8; Field, 2018). No distinction was made between the BP and the EP hazards as it was no primary aim of this study and due to the small number of BP trials and the small sample size.

## Results

A detailed representation of the gaze behavior and response rate data of DCD and TD participants per hazard, can be found in Supplementary Table 2.

### Cycling Experience and Perceived Cycling Abilities

Results from the questionnaire indicated no difference in the age that children started to learn how to cycle between the DCD group (5.13 ± 1.46) and the TD group [4.44 ± 1.01; *t*(15) = −1.129, *p* = 0.277, *d* = 0.671]. However, the participants with DCD reported to have mastered to motor skill of biking significantly later (6.63 ± 2.07) than the TD participants [4.83 ± 1.00; *t*(15) = −2.320, *p* = 0.035, *d* = 1.792]. One participant with DCD reported that she was, at the time of the test, still not able to safely cycle in traffic as an adult. The remaining participants with DCD indicated they were able to safely cycle in traffic since the age of 9.86 ± 3.29, which did not significantly differ from that of the TD group [10.44 ± 1.74; *t*(8.593) = 0.428, *p* = 0.679, *d* = −0.338]. The TD individuals reported to have, on average, more years of cycling experience (19.67 ± 4.03) than the DCD group [15.25 ± 5.73; *t*(15) = 1.857, *p* = 0.083, *d* = −1.096]. Furthermore, TD participants appeared to cycle more often (4.48 ± 2.56 times per week) compared to their DCD counterparts (2.93 ± 2.75), but no significant effect was detected [*t*(15) = 1.199, *p* = 0.249, *d* = −0.603]. As to the perception of cycling ability, the participants with DCD (3.25 ± 1.04) perceived themselves as significantly less proficient cyclists on the 5-point Likert scale than the TD group (4.56 ± 0.53; Mann–Whitney U = 10.500, *p* = 0.010, *d* = −2.477). Finally, their perception of their anticipation skills in traffic when cycling is significantly below (3.38 ± 0.92) that of the TD participants (4.56 ± 0.53; Mann–Whitney U = 10.500, *p* = 0.010, *d* = −2.240).

### Hazard Perception Test

#### Gaze Behavior

Descriptive statistics for of the gaze behavior variables are presented in Table 2. Interestingly, in 21.6% of all hazards presented to the DCD participants, the hazard was not fixated at all, whereas this was only the case in 6.5% of the hazards in the TD group. The DCD group used significantly less fixations compared to the TD group and the participants with DCD appeared to fixate the hazard significantly later than the TD group. No differences were found between the groups for the average duration of all fixations or the duration of the first fixation on the hazard. However, dwell time on the hazards did differ between the groups, with the individuals with DCD spending less time fixating the hazards than the TD participants.

**TABLE 2:** Average *z*-score values for the gaze behavior variables (mean ± SD).

	DCD	TD	*t*	df	*p*	Cohen’s *d*
Number of fixations	−0.57 ± 0.73	0.00 ± 0.46	3.211	15	0.006*	−2.235**
Timing first fixation	0.65 ± 0.58	0.03 ± 0.41	−2.606	15	0.020*	1.538**
Average fixation duration	0.18 ± 0.58	−0.01 ± 0.58	−0.665	15	0.516	0.325
First fixation duration	0.22 ± 0.50	−0.03 ± 0.39	−1.185	15	0.254	0.656
Dwell time	−0.63 ± 0.49	0.00 ± 0.46	2.727	15	0.016*	−1.367**

*Significant: p < 0.05. **Large effect size: | d| > 0.8.

#### Response Rate and Reaction Time

Individuals with DCD clicked for 85.29 ± 16.03 percent of the hazards, which did not differ from the response rate of the TD participants [76.47 ± 16.38; *t*(15) = −1.120, *p* = 0.280, *d* = 0.539]. In addition, the participants with DCD tended to make more extra clicks (5.00 ± 4.66) than the TD participants (2.11 ± 2.80), however, this difference was not significant [*t*(15) = −1.571, *p* = 0.137, *d* = 1.030]. On most of the hazards, individuals with DCD seem to respond later (*z*-score: 0.64 ± 1.32) compared to the TD group (0.11 ± 0.66). However, despite a large effect size, there was no significant difference on this variable [*t*(15) = −1.071, *p* = 0.301, *d* = 0.807].

#### Perception of Safety

The participants with DCD rated the traffic situations in the videos as significantly more dangerous (3.19 ± 0.44) compared to the TD individuals [2.20 ± 0.77; *t*(15) = −3.191, *p* = 0.006, *d* = 1.284].

## Discussion

The current study explored if young adults with DCD perceive and react to traffic hazards differently than TD participants. Individuals with DCD fixated the hazards later than the TD participants, made fewer fixations on the hazards, and spent less time fixating them. However, no significant differences in response rate or reaction time were found.

The questionnaire on cycling experience and perceived cycling abilities revealed that individuals with DCD took more time to learn to ride a bike. However, they also indicated to be able to safely cycle in traffic at around the same age as the TD individuals. Furthermore, the participants with DCD had less experience in cycling and they rated themselves as significantly less proficient cyclists than their TD counterparts. Also, the DCD group rated its anticipation skills to be worse than the TD group. This corresponds with the findings on road crossing, where, over half of the adult respondents with DCD indicated to be not or only somewhat confident in road crossing (Wilmut and Purcell, 2020). It therefore seems reasonable to assume that for individuals with DCD, the issues that have been reported in road crossing will also persist in cycling and other modes of transportation.

It is interesting to note that while gaze behavior was different, i.e., later fixation and shorter dwell times on the hazards in the adults with DCD, no difference was found in the reaction to the hazard. The implication is that the time between the first fixation and reaction to the hazard was longer in TD individuals than in their counterparts with DCD. Judging traffic requires a continuous cycle of perception, appraisal, and prediction of a multitude of visual cues. The advanced first fixation and longer dwell times of the TD adults, therefore, suggest a better “situational awareness” in this group, with a more goal-directed visual search strategy (i.e., toward potential hazards) and more revisits of regions that may be or become potentially hazardous. The advantage of early recognition of an object or event that will become a hazard, is that one has time to anticipate. However, the lack of differences in reaction time between DCD and TD indicates that individuals with DCD seem to not have problems with recognizing an object or event as dangerous and reacting to it. The finding that they pick up hazards later and are less attentive to what may develop as hazardous later on, may suggest poor predictive abilities in hazard perception in DCD. The implication is that these individuals would need to react to dangers, rather than being in a position where they can anticipate. Although no differences were found in the number of extra clicks between the groups, the higher total number of clicks in the DCD group may suggest that they react more to anything that may be hazardous, rather than anticipating actual hazards. It is likely that this will contribute to an increased perception of risk in DCD, as found in this study and consistent with Wilmut and Purcell (2020). The differences in gaze behavior may also be influenced by other factors. First, individuals with DCD are known to demonstrate oculomotor problems which might have impacted on the saccadic behavior in this task (Sumner et al., 2018). These oculomotor deficits have been found to surface in other daily tasks. For example, Wilmut et al. (2006) found a delayed initiation of eye movements in a sequential pointing task, and, in catching, children with DCD require more time to fixate and track the ball (Licari et al., 2018). Secondly, the gaze behavior in the hazard perception task may also be reflective of an increased need to focus on the path, as found in adults with DCD while walking (Warlop et al., 2020). If this would also be the visual strategy used in cycling in individuals with DCD, it may have distracted them from detecting hazards, as these usually occur further down the road. This suggestion should be subject to further research. Thirdly, it should be noted that the videos used in the current test were recorded on TD adults. As individuals with DCD are known to adopt compensatory strategies, they might also cycle slower than TD individuals. As a consequence, the videos shown in the current test could reflect a “normal” optical flow for a TD individual, whereas it displayed a faster flow than what individuals with DCD are used to, which might have led to a less appropriate gaze behavior in the DCD group. Fourthly, the difference in gaze behavior might also be caused by a lack of cycling experience in the DCD group. Young inexperienced cyclists were found to have delayed first fixations and slower reaction times to hazards than experienced adults (Vansteenkiste et al., 2016; Zeuwts et al., 2016), so the “immature” gaze behavior of individuals with DCD might be a reflection of a lack of experience with traffic too. Finally, the altered gaze behavior may be related to the cognitive requirements of the task. The hazard perception test may be more demanding for individuals with DCD, who are known to have poorer working memory capacity (Alloway, 2011). Similar results, with later fixations and reduced fixation times on the hazards, were found in hazard perception tests under increased cognitive load (Wood et al., 2016). In contrast to our study, this resulted in reduced hazard perception performance. It is possible that with higher cognitive load, with for example addition of a motor component, the differences in gaze behavior found in this task may be accompanied by reduced hazard perception performances in individuals with DCD.

In this study, we investigated the hazard perception skills of DCD, while neutralizing the motor challenges related to cycling. The benefit of this approach is that it enabled us to demonstrate that in terms of perception, comprehension, and projection of visual cues and events alone, individuals with DCD already experience problems. In combination with their motor problems, this may lead to a higher risk of accidents during cycling in traffic. The disadvantage of the current paradigm is of course that the motor response was limited to a mouse click. In cycling, leg movements need to be coordinated with accurate arm and hand movements, while balancing on the bike and responding to dynamic traffic situations. This does not only add to the motor difficulty of the task, but also increases the cognitive load, which may negatively impact both the gaze behavior and the reactions toward hazards in traffic (Wood et al., 2016). To get a better understanding of the problems in cycling with DCD, future research should consider more complex tasks with a combination of both the perceptual and motor aspect of the task. Also, while our findings are supported with large effect sizes, it must be acknowledged that the sample size is relatively small. As DCD is a heterogeneous disorder, we recommend future studies to include more participants and to broaden the age range of the sample to children as well.

## Conclusion

In conclusion, the gaze behavior of young adults with DCD differs from that of TD individuals in a hazard perception task, characterized by a delayed fixation on hazards, fewer fixations and less time spent fixating the hazards. It is unclear whether this altered gaze behavior causes an increased risk for accidents, as no differences were found in the reactions to the hazards. However, it does indicate that not only the motor difficulties should be taken into consideration in therapy for cycling. This is all the more important as the perceived risk experienced by the adults with DCD may lead to withdrawal of active participation in traffic. As cycling is part of a healthy lifestyle and an increasingly important means of transport, future studies should investigate interventions targeting the specific problems highlighted in this study.

## Data Availability Statement

The raw data supporting the conclusions of this article will be made available by the authors, without undue reservation.

## Ethics Statement

The studies involving human participants were reviewed and approved by the Ethics Committee of Ghent University Hospital. The patients/participants provided their written informed consent to participate in this study.

## Author Contributions

PV, FD, and ML contributed to the conception of the study and critically reviewed the manuscript. PV designed the study protocol. GW collected and analyzed the data and wrote the first draft of the manuscript. All authors approved the final version of the manuscript for submission.

## Conflict of Interest

The authors declare that the research was conducted in the absence of any commercial or financial relationships that could be construed as a potential conflict of interest.

## References

[B1] Alloway T. P. (2011). A comparison of working memory profiles in children with ADHD and DCD. *Child Neuropsychol.* 17 483–494. 10.1080/09297049.2011.553590 21424949

[B2] American Psychiatric Association (2013). *Diagnostic and Statistical Manual of Mental Disorders (DSM-5 R).* Arlington, VA: American Psychiatric Publishing.

[B3] Clancy T. A. Rucklidge J. J. Owen D. (2006). Road-crossing safety in virtual reality: a comparison of adolescents with and without ADHD. *J. Clin. Child Adolesc. Psychol*. 35 203–215. 10.1207/s15374424jccp3502_4 16597216

[B4] Cowan G. Earl R. Falkmer T. Girdler S. Morris S. L. (2018). Fixation patterns of individuals with and without Autism spectrum disorder : Do they differ in shared zones and in zebra crossings ? *J. Transp. Health* 8 112–122. 10.1016/j.jth.2017.12.001

[B5] Crundall D. Chapman P. Trawley S. Collins L. van Loon E. Andrews B. (2012). Some hazards are more attractive than others: drivers of varying experience respond differently to different types of hazard. *Accid. Anal. Prev.* 45 600–609. 10.1016/j.aap.2011.09.049 22269547

[B6] Deconinck F. J. A. De Clercq D. Savelsbergh G. J. P. Van Coster R. Oostra A. Dewitte G. (2006a). Adaptations to task constraints in catching by boys with DCD. *Adapt. Phys. Activ. Q.* 23 14–30. 10.1123/apaq.23.1.14

[B7] Deconinck F. J. A. De Clercq D. Savelsbergh G. J. P. Van Coster R. Oostra A. Dewitte G. (2006b). Visual contribution to walking in children with developmental coordination disorder. *Child Care Health Dev.* 32 711–722. 10.1111/j.1365-2214.2006.00685.x 17018046

[B8] Du W. Wilmut K. Barnett A. L. (2015). Level walking in adults with and without developmental coordination disorder: an analysis of movement variability. *Hum. Mov. Sci.* 43 9–14. 10.1016/j.humov.2015.06.010 26149451

[B9] Endsley M. R. (1995). Toward a theory of situation awareness in dynamic systems. *Hum. Fact.* 37 32–64. 10.1518/001872095779049543 27006588

[B10] Field A. (2018). *Discovering Statistics using IBM SPSS Statistics*, 5th Edn. London: SAGE Publications.

[B11] Henderson S. E. Sugden D. A. Barnett A. L. (2010). *Movement Assessment Battery for Children-2.* Amsterdam: Pearson Education Limited.

[B12] Kirby A. Rosenblum S. (2008). *The Adult Developmental Coordination Disorder/Dyspraxia Checklist (ADC) for Further and Higher Education.* Newport: Dyscovery Centre at the University of Wales. Available online at: http://psychology.research.southwales.ac.uk/research/developmental-psychology/amanda-kirby/index.html

[B13] Kirby A. Edwards L. Sugden D. (2011a). Driving behaviour in young adults with developmental coordination disorder. *J. Adult Dev.* 18 122–129. 10.1007/s10804-011-9120-4

[B14] Kirby A. Edwards L. Sugden D. (2011b). Emerging adulthood in developmental co-ordination disorder: parent and young adult perspectives. *Res. Dev. Disabil.* 32 1351–1360. 10.1016/j.ridd.2011.01.041 21334175

[B15] Licari M. K. Reynolds J. E. Tidman S. Ndiaye S. Sekaran S. N. Reid S. L. (2018). Visual tracking behaviour of two-handed catching in boys with developmental coordination disorder. *Res. Dev. Disabil.* 83 280–286. 10.1016/j.ridd.2018.07.005 30097307

[B16] Purcell C. Wann J. P. Wilmut K. Poulter D. (2012). Reduced looming sensitivity in primary school children with developmental co-ordination disorder. *Dev. Sci.* 3 299–306. 10.1111/j.1467-7687.2011.01123.x 22490171

[B17] Purcell C. Wilmut K. Wann J. P. (2017). Human movement science the use of visually guided behaviour in children with developmental coordination disorder when crossing a virtual road. *Hum. Mov. Sci.* 53 37–44. 10.1016/j.humov.2016.11.007 27939726

[B18] Sumner E. Hutton S. B. Kuhn G. Hill E. L. (2018). Oculomotor atypicalities in developmental coordination disorder. *Dev. Sci.* 21:e12501. 10.1111/desc.12501 27753223 PMC5763390

[B19] Tsai C. L. Chang Y. K. Hung T. M. Tseng Y. T. Chen T. Z. (2012). The neurophysiological performance of visuospatial working memory in children with developmental coordination disorder. *Dev. Med. Child Neurol.* 54 1114–1120. 10.1111/j.1469-8749.2012.04408.x 22978755

[B20] Vansteenkiste P. Zeuwts L. Cardon G. Lenoir M. (2016). A hazard-perception test for cycling children: an exploratory study. *Transp. Res. Part F* 41 182–194. 10.1016/j.trf.2016.05.001

[B21] Warlop G. Vansteenkiste P. Lenoir M. Van Causenbroeck J. Deconinck F. J. A. (2020). Gaze behaviour during walking in young adults with developmental coordination disorder. *Hum. Mov. Sci.* 71:102616. 10.1016/j.humov.2020.102616 32452432

[B22] Wetton M. A. Hill A. Horswill M. S. (2011). The development and validation of a hazard perception test for use in driver licensing. *Accid. Anal. Prev.* 43 1759–1770. 10.1016/j.aap.2011.04.007 21658504

[B23] Wilmut K. Purcell C. (2020). The lived experience of crossing the road when you have developmental coordination disorder (DCD): the perspectives of parents of children with DCD and adults with DCD. *Front. Psychol.* 11:587042. 10.3389/fpsyg.2020.587042 33329244 PMC7710519

[B24] Wilmut K. Purcell C. (2021). The nature of the risk faced by pedestrians with neurodevelopmental disorders: a systematic review. *Accid. Anal. Prev.* 149:105886. 10.1016/j.aap.2020.105886 33248701

[B25] Wilmut K. Byrne M. Barnett A. L. (2013). Reaching to throw compared to reaching to place: a comparison across individuals with and without developmental coordination disorder. *Res. Dev. Disabil.* 34 174–182. 10.1016/j.ridd.2012.07.020 22944258

[B26] Wilmut K. Wann J. P. Brown J. H. (2006). Problems in the coupling of eye and hand in the sequential movements of children with developmental coordination disorder. *Child Care Health Dev.* 32 665–678. 10.1111/j.1365-2214.2006.00678.x 17018042

[B27] Wilson P. H. McKenzie B. E. (1998). Information processing deficits associated with developmental coordination disorder: a meta-analysis of research findings. *J. Child Psychol. Psychiatry* 39 829–840. 10.1017/S0021963098002765 9758192

[B28] Wilson P. H. Ruddock S. Smits-Engelsman B. Polatajko H. Blank R. (2013). Understanding performance deficits in developmental coordination disorder: a meta-analysis of recent research. *Dev. Med. Child Neurol.* 55 217–228. 10.1111/j.1469-8749.2012.04436.x 23106668

[B29] Wood G. Hartley G. Furley P. A. Wilson M. R. (2016). Working memory capacity, visual attention and hazard perception in driving. *J. Appl. Res. Mem. Cogn.* 5 454–462. 10.1016/j.jarmac.2016.04.009

[B30] Zeuwts L. H. R. H. Vansteenkiste P. Deconinck F. J. A. Cardon G. Lenoir M. (2016). Hazard perception in young cyclists and adult cyclists. *Accid. Anal. Prev.* 105 64–71. 10.1016/j.aap.2016.04.034 27174373

