# Supplementary material for: Young Adults With Developmental Coordination Disorder Adopt a Different Visual Strategy During a Hazard Perception Test for Cyclists
Source: Front Psychol. 2021 Apr 14;12:665189. doi: 10.3389/fpsyg.2021.665189 (PMC8079720; doi:10.3389/fpsyg.2021.665189)
Supplement: Supplementary file 1 [file Data_Sheet_1.PDF]

Supplementary table 1. Description and details of the clips and hazards included in the hazard perception test.

CLIP	HAZARD	DESCRIPTION	HAZARD	BP/EP	DURATION (s)
1	1	While cycling along a cycling path, a pedestrian walking on the right, suddenly crosses the path and a car approaches from a side street on the right	The pedestrian	BP	5.84
	2		The car, stopping halfway on the cycling path	EP	8.21
2	1	While cycling on the road with parked cars on the right, a pedestrians walks on the right of the cars and suddenly crosses the street from behind van in front of the cyclist	The pedestrian	EP	2.20
3	1	While cycling on a cycling path with parked cars on the left, a pedestrian crosses the cycling path from between two cars in front of the cyclist	The pedestrian	EP	2.45
4	1	While cycling on the road along parked cars on the right, a man suddenly opens his car door and steps out of his car	The man coming out of his car	EP	10.45
5	1	While cycling on the road and crossing a street in the right, a car approaches fast from the left in front of the cyclist and turns to the street on the right.	The car	EP	2.82
6	1	The cyclist is cycling along a cycling path and crossing a side street on the right from which a car is approaching	The car	EP	3.48
7	1	While cycling on the cycling path, the cyclist had to enter the car road due to a container blocking the path	A container in the middle of the cycling path	BP	3.31
8	1	The cyclist crosses an intersection while a pedestrian and a cyclist, coming from the right, cross the intersection	The pedestrian coming from behind a car on the right on a crosswalk	EP	4.24
	2		The cyclist	EP	2.07
9	1	While cycling between tram lines on the left and cars on the right, a pedestrian suddenly appears from behind a car	The pedestrian, waiting to cross until the cyclist has passed	EP	1.93
10	1	While cycling on a cycling path, a pedestrian approaches the path from the right and crosses it in front of the cyclist	The pedestrian	BP	1.53
11	1	While cycling in a busy street, the cyclist crosses two intersections with on the first a car approaching from the right and on the second a car crossing from the left. Two pedestrians cross the street.	The car approaching from the right on the first intersection	EP	2.87
	2		A pedestrian suddenly walking onto the street from the right	BP	2.08
	3		A pedestrian walking along the pavement on the left and crossing the street in front of the cyclist	BP	2.17
	4		A car suddenly crossing the second intersection from the left, the cyclist had priority but had to stop for the car	EP	2.24
12	1	While cycling alongside parked cars on the right, a pedestrian suddenly wants to cross the street from behind a van	The pedestrian, waiting to cross until the cyclist has passed	EP	1.68

* BP = behavioral prediction, EP = environmental prediction
