# Supplementary material for: Young Adults With Developmental Coordination Disorder Adopt a Different Visual Strategy During a Hazard Perception Test for Cyclists
Source: Front Psychol. 2021 Apr 14;12:665189. doi: 10.3389/fpsyg.2021.665189 (PMC8079720; doi:10.3389/fpsyg.2021.665189)
Supplement: Supplementary file 2 [file Data_Sheet_2.PDF]

Supplementary table 2. Detailed gaze behavior and response data of the HP-test (mean ± SD), including mean and SD of z-scores of the DCD group and Cohen's d for each hazard separately.

		Clip 1		Clip 2		Clip 3		Clip 4		Clip 5		Clip 6	
		Hazard 1		Hazard 1		Hazard 1		Hazard 1		Hazard 1		Hazard 1	
		DCD	TD	DCD	TD	DCD	TD	DCD	TD	DCD	TD	DCD	TD
Number of fixations	Raw	4.25 ± 2.25	4.00 ± 1.00	2.25 ± 2.05	2.67 ± 1.73	1.88 ± 1.46	2.89 ± 2.15	3.63 ± 1.19	4.78 ± 1.64	3.75 ± 2.12	2.78 ± 1.30	2.50 ± 1.51	3.67 ± 1.50
	Z-score	0.25 ± 2.25		-0.24 ± 1.19		-0.47 ± 0.68		-0.70 ± 0.72		0.75 ± 1.63		-0.78 ± 1.01	
	D	0.25		-0.241		-0.472		-0.702		0.747		-0.778	
Average fixation duration	Raw	374.75 ± 106.52	389.91 ± 109.58	321.60 ± 129.32	325.22 ± 114.06	493.72 ± 223.33	436.93 ± 165.91	514.62 ± 212.01	517.53 ± 173.18	394.57 ± 220.91	361.56 ± 122.73	393.30 ± 120.54	405.29 ± 136.39
	Z-score	-0.14 ± 0.97		-0.03 ± 1.13		0.34 ± 1.35		-0.02 ± 1.22		0.27 ± 1.80		-0.09 ± 0.88	
	D	-0.138		-0.032		0.342		- 0.017		0.269		-0.088	
First fixation duration	Raw	362.25 ± 186.21	436.80 ± 229.85	412.74 ± 192.99	274.07 ± 175.62	474.01 ± 207.14	514.98 ± 176.40	583.02 ± 232.10	521.67 ± 311.76	413.90 ± 303.53	392.63 ± 145.38	543.24 ± 250.38	494.91 ± 177.74
	Z-score	-0.32 ± 0.81		0.79 ± 1.10		-0.23 ± 1.17		0.20 ± 0.74		0.15 ± 2.09		0.27 ± 1.41	
	D	-0.324		0.790		-0.232		0.197		0.146		0.272	
Dwell time	Raw	1531.47 ± 798.02	1546.39 ± 560.11	632.86 ± 488.71	905.09 ± 667.12	820.16 ± 472.58	1166.40 ± 600.55	1760.15 ± 603.85	2434.13 ± 1074.17	1220.87 ± 414.15	1028.24 ± 590.81	1041.49 ± 741.85	1357.90 ± 409.88
	Z-score	-0.03 ± 1.42		-0.41 ± 0.73		-0.58 ± 0.79		-0.63 ± 0.56		0.33 ± 0.70		-0.77 ± 1.81	
	D	-0.027		-0.408		-0.577		-0.627		0.326		-0.772	
Timing first fixation	Raw	207.16 ± 923.15	67.16 ± 920.65	842.92 ± 386.00	470.19 ± 487.70	1317.86 ± 567.33	651.68 ± 489.88	927.32 ± 1013.90	400.45 ± 1174.60	224.13 ± 231.17	218.45 ± 259.95	774.77 ± 445.97	638.02 ± 244.56
	Z-score	0.15 ± 1.00		0.76 ± 0.79		1.36 ± 1.16		0.45 ± 0.86		0.02 ± 0.89		0.56 ± 1.82	
	D	0.152		0.764		1.360		0.449		0.022		0.559	
Response rate	Raw	1.00 ± 0.00	1.00 ± 0.00	0.88 ± 0.35	0.88 ± 0.33	1.00 ± 0.00	1.00 ± 0.00	0.88 ± 0.35	0.67 ± 0.50	0.63 ± 0.52	0.56 ± 0.53	0.88 ± 0.35	1.00 ± 0.00
	Z-score												
	D			-0.042				0.417		0.132			
Reaction time	Raw	1304.34 ± 792.73	374.28 ± 347.85	763.61 ± 190.29	660.11 ± 205.73	1112.22 ± 637.39	810.19 ± 250.10	880.85 ± 1198.07	913.95 ± 581.82	-622.88 ± 1436.02	101.28 ± 540.47	1012.73 ± 356.35	759.34 ± 179.67
	Z-score	2.67 ± 2.28		0.50 ± 0.92		1.21 ± 2.55		-0.06 ± 2.06		-1.34 ± 2.66		1.41 ± 1.98	
	D	2.67		0.503		1.208		-0.057		-1.340		1.410	

		Clip 7		Clip 7		Clip 8		Clip 9		Clip 10		Clip 10	
		Hazard 1		Hazard 2		Hazard 1		Hazard 1		Hazard 1		Hazard 2	
		DCD	TD	DCD	TD	DCD	TD	DCD	TD	DCD	TD	DCD	TD
Number of fixations	Raw	3.13 ± 3.40	4.22 ± 1.56	5.50 ± 2.14	4.11 ± 1.96	1.38 ± 1.30	3.78 ± 1.20	2.88 ± 1.46	6.44 ± 3.57	1.88 ± 1.13	3.56 ± 1.51	1.13 ± 1.36	3.11 ± 1.83
	Z-score	-0.70 ± 2.17		0.71 ± 1.09		-2.00 ± 1.08		-1.00 ± 0.41		-1.11 ± 0.75		-1.08 ± 0.74	
	D	-0.702		0.707		-1.999		-0.999		-1.114		-1.083	
Average fixation duration	Raw	455.11 ± 202.55	492.89 ± 235.05	195.68 ± 57.85	198.79 ± 52.26	496.32 ± 326.32	271.66 ± 71.80	487.32 ± 242.40	352.99 ± 182.31	317.56 ± 128.37	289.06 ± 122.63	581.05 ± 369.38	333.96 ± 176.54
	Z-score	-0.16 ± 0.86		-0.06 ± 1.11		3.13 ± 4.54		0.74 ± 1.33		0.23 ± 1.05		1.40 ± 2.09	
	D	-0.161		-0.059		3.129		0.737		0.232		1.400	
First fixation duration	Raw	490.89 ± 150.30	844.69 ± 495.59	148.79 ± 45.57	217.42 ± 76.45	497.71 ± 361.95	341.61 ± 165.19	615.51 ± 548.32	353.12 ± 217.12	368.86 ± 312.89	245.00 ± 153.81	578.95 ± 385.68	321.57 ± 202.02
	Z-score	-0.71 ± 0.30		-0.90 ± 0.60		0.94 ± 2.19		1.21 ± 2.53		0.81 ± 2.03		1.27 ± 1..91	
	D	-0.714		-0.898		0.945		1.208		0.805		1.274	
Dwell time	Raw	1079.94 ± 817.93	1932.54 ± 741.73	1002.72 ± 239.74	802.10 ± 336.45	578.68 ± 464.79	999.95 ± 345.71	1289.97 ± 699.82	2024.82 ± 883.13	713.58 ± 700.98	1003.57 ± 574.82	496.37 ± 459.77	962.83 ± 527.77
	Z-score	-1.15 ± 1.10		0.60 ± 0.71		-1.22 ± 1.34		-0.83 ± 0.79		-0.50 ± 1.22		-0.88 ± 0.87	
	D	-1.149		0.596		-1.219		-0.832		-0.504		-0.884	
Timing first fixation	Raw	836.10 ± 1461.58	394.38 ± 751.89	521.16 ± 144.13	795.43 ± 424.48	370.50 ± 448.46	186.97 ± 246.96	-164.15 ± 1274.54	-896.94 ± 1235.93	1105.56 ± 444.79	949.73 ± 250.19	1153.76 ± 711.55	956.96 ± 699.66
	Z-score	0.59 ± 1.95		-0.65 ± 0.34		0.74 ± 1.82		0.59 ± 1.03		0.62 ± 1.78		0.28 ± 1.02	
	D	0.587		-0.646		0.743		0.593		0.623		0.281	
Response rate	Raw	0.88 ± 0.35	0.67 ± 0.50	1.00 ± 0.00	0.67 ± 0.50	0.88 ± 0.35	0.78 ± 0.44	0.88 ± 0.35	0.78 ± 0.44	0.75 ± 0.46	0.78 ± 0.44	0.88 ± 0.35	0.56 ± 0.53
	Z-score												
	D	0.417		0.667		0.220		0.220		-0.063		0.606	
Reaction time	Raw	1600.62 ± 1051.75	1550.94 ± 578.01	1135.24 ± 348.20	879.79 ± 166.64	197.22 ± 858.69	454.70 ± 611.91	-1123.83 ± 1657.54	-801.99 ± 1737.25	1475.43 ± 1101.42	1391.25 ± 1013.56	1208.41 ± 1054.85	536.23 ± 939.19
	Z-score	0.09 ± 1.82		1.53 ± 2.09		-0.42 ± 1.40		-0.19 ± 0.95		0.08 ± 1.09		0.72 ± 1.12	
	D	0.086		1.533		-0.421		-0.185		0.083		0.716	

		Clip 10		Clip 10		Clip 11		Clip 12		Clip 12	
		Hazard 3		Hazard 4		Hazard 1		Hazard 1		Hazard 2	
		DCD	TD	DCD	TD	DCD	TD	DCD	TD	DCD	TD
Number of fixations	Raw	1.00 ± 1.07	3.22 ± 1.64	6.63 ± 3.78	8.11 ± 2.47	2.00 ± 1.07	4.00 ± 1.94	6.38 ± 3.38	7.22 ± 1.64	19.25 ± 4.83	18.78 ± 3.03
	Z-score	-1.35 ± 0.65		-0.60 ± 1.53		-1.03 ± 0.55		-0.52 ± 2.06		0.16 ± 1.59	
	D	-1.354		-0.601		-1.033		-0.516		0.156	
Average fixation duration	Raw	656.58 ± 322.24	361.11 ± 167.02	252.04 ± 64.48	262.20 ± 119.66	296.68 ± 154.12	323.12 ± 100.61	380.11 ± 103.47	547.17 ± 181.93	341.73 ± 80.60	396.00 ± 117.06
	Z-score	1.77 ± 1.93		-0.08 ± 0.54		-0.26 ± 1.53		-0.92 ± 0.57		-0.46 ± 0.69	
	D	1.769		-0.085		-0.263		-0.918		-0.464	
First fixation duration	Raw	662.44 ± 445.71	302.52 ± 128.91	234.73 ± 140.09	281.74 ± 196.49	246.10 ± 162.54	333.63 ± 124.89	575.58 ± 343.44	593.19 ± 373.70	434.77 ± 222.35	329.32 ± 159.46
	Z-score	2.79 ± 3.46		-0.24 ± 0.71		-0.70 ± 1.30		-0.05 ± 0.92		0.66 ± 1.39	
	D	2.792		-0.239		-0.701		-0.047		0.661	
Dwell time	Raw	634.13 ± 701.72	1210.79 ± 807.63	1582.76 ± 774.09	2022.85 ± 852.32	583.35 ± 415.09	1158.46 ± 344.53	2471.26 ± 1659.62	3900.29 ± 1284.80	6273.45 ± 623.04	7180.92 ± 1509.48
	Z-score	-0.71 ± 0.87		-0.52 ± 0.91		-1.67 ± 1.20		-1.11 ± 1.29		-0.60 ± 0.41	
	D	-0.714		-0.516		-1.669		-1.112		-0.601	
Timing first fixation	Raw	1095.41 ± 658.01	889.71 ± 554.47	1754.83 ± 503.55	1007.03 ± 690.30	836.61 ± 425.39	302.82 ± 301.93	939.42 ± 2008.15	102.77 ± 1118.12	1093.25 ± 1468.15	434.12 ± 800.05
	Z-score	0.37 ± 1.19		1.08 ± 0.73		1.77 ± 1.41		0.75 ± 1.80		0.82 ± 1.84	
	D	0.371		1.083		1.768		0.748		0.824	
Response rate	Raw	0.63 ± 0.52	0.67 ± 0.50	0.88 ± 0.35	1.00 ± 0.00	0.75 ± 0.46	0.33 ± 0.50	0.75 ± 0.46	0.67 ± 0.50	1.00 ± 0.00	1.00 ± 0.00
	Z-score										
	D	-0.083				0.833		0.167			
Reaction time	Raw	1401.18 ± 937.46	1088.22 ± 531.85	1181.33 ± 1740.51	1449.20 ± 349.90	42.35 ± 1739.51	-1096.39 ± 1084.88	1864.70 ± 1303.51	1481.72 ± 952.57	1596.50 ± 1681.26	1594.50 ± 1080.96
	Z-score	0.59 ± 1.76		-0.77 ± 4.97		1.05 ± 1.60		0.40 ± 1.37		0.00 ± 1.56	
	D	0.588		-0.766		1.050		0.402		0.002	
